# An Atypical Case of Morbid Obesity, Presenting With Deep Vein Thrombosis and Pulmonary Embolism

**DOI:** 10.7759/cureus.9650

**Published:** 2020-08-11

**Authors:** Akshit Chitkara, Abdulhassan Saad

**Affiliations:** 1 Internal Medicine, Beaumont Hospital, Dearborn, USA

**Keywords:** extreme obesity, deep vein thrombosis (dvt), pulmonary embolism (pe), type 2 diabetes

## Abstract

Extreme obesity or Class III obesity is defined as a body mass index (BMI) greater than 40 kg/m^2^ and is invariably associated with a marked increase in morbidity and mortality. Our patient is a 36-year-old male wrestler, with a BMI of 63.53 kg/m^2^, and has been suffering from recurrent deep vein thrombosis (DVT), pulmonary embolism, osteoarthritis of both knees, obstructive sleep apnea, insulin-dependent type ll diabetes mellitus, fungal infections, pulmonary hypertension, neuropathy, and foot ulcers. He has been subjected to multiple surgeries and interventions, primarily because of his profession. He presents to us with pain in his chest and in his left leg for the past two days. Extreme obesity is a global health problem and has acquired the level of a pandemic. This case emphasizes the role of early detection and management of extreme obesity and its complications.

## Introduction

Obesity is an increasingly prevalent and complex medical condition, that contributes to a variety of chronic disorders [1, 2]. Class III (extreme) obesity is defined as a BMI of 40 kg/m^2^ or higher, and its prevalence is increasing globally at a swift pace, across all sections of the society [3]. It has become a major health concern because of the associated morbidity and mortality [4-6]. Certain adverse environmental conditions, such as high‐fat diets, sedentary lifestyles, increasing age, and low socioeconomic status along with genetic predisposition, have been reported to be predictive of weight gain. These can help physicians identify which patients are obese or at risk of developing obesity.

The increased body weight and the central adiposity along with hyperlipidemia and other disturbed parameters may lead to multiple disorders such as insulin resistance, metabolic syndrome, type ll diabetes mellitus, hypertension, increased cardiometabolic risk along with subsequent renal and neurological involvement and risk of recurrent infections. There is a significant positive association between degenerative processes in the musculoskeletal system and the level of obesity [7-9].

## Case presentation

We present a case of a 36-year-old male wrestler, presented with pain in his chest and in his left leg for the past two days. He also complained of pain in the big toe. There is no history of palpitation, dyspnea, sweating, or cough. No fever or headache. There is no history of trauma of lower limbs or any neurological deficit. There is no urinary or bowel trouble. There is no history of vomiting, diarrhea, or pain in abdomen. The patient is a chronic cigarette smoker with a history of two packets of cigarettes per week for the past 20 years. There is no history of recreational drug use.

The patient had a history of recurrent deep vein thrombosis, pulmonary embolism (PE), obstructive sleep apnea, insulin-dependent type ll diabetes mellitus, and candidal balanitis. There is a history of multiple surgeries in the past. The patient had undergone left elbow surgery, left knee surgery, right ankle surgery, and foot surgery, due to injuries caused by his profession. The patient has mild pitting edema in both feet.

On examination, the patient is extremely obese with a weight of 456 lbs., a height of 188 cm, and a BMI of 63.53 kg/m^2^. His blood pressure is 99/56 mm of Hg, a temperature of 98.6 F, respiratory rate of 16 per minute, and oxygen saturation of 98%. The patient is fully conscious and well oriented. There is no anemia, cyanosis, or jaundice.

Doppler report of the lower limbs showed bilateral deep vein thrombosis (DVT) of popliteal veins and left common femoral vein, otherwise normal Doppler flow compressibility of profunda femoris and superficial femoral veins. Based on the clinical, laboratory, and the Doppler report, the patient was diagnosed with pulmonary embolism with bilateral DVT of popliteal veins and left common femoral vein (Table 1).

**Table 1:** Lab Results APTT: Activated partial thromboplastin time; PT: Prothrombin time.

Name of Test	Patient Value	Reference Range
Fasting serum glucose	299 mg per dL	70-110 mg per dL
Serum Sodium	134 mEq per L	136-145 mEq per L
Urine specific gravity	1.032	1.010-1.025
Beta-hydroxybutyrate	2.09 mmol per L	<0.5 mmol per L
APTT	77.2 seconds	25-40 seconds
PT	15.9 seconds	11-15 seconds
D-dimer	816 nano-gram per ml	<250 nano-gram per ml

Treatment

The patient was admitted to the inpatient unit. After consulting cardiologist, IV heparin was started right away for DVT with pulmonary embolism.

He was also prescribed supportive therapy for controlling blood glucose and obesity, in consultation with an endocrinologist and dietician. The patient was advised for lifestyle modifications such as losing weight and staying active, to manage DVT, obesity and co-morbidities.

## Discussion

Obesity is increasing rapidly around the world, in both adults and children. Studies have shown that obese individuals have nearly twice the risk of both pulmonary embolism and DVT and obese patients less than 40 years of age have nearly a five-fold risk than those who are not obese [10]. Extreme obesity is a global health problem and has acquired the level of a pandemic. This case emphasizes the role of early detection and management of extreme obesity and its complications, as there is a strong observational association between obesity and DVT with or without PE [10].

Obesity is not only caused by a positive balance of daily calorie intake, but there are multiple factors responsible for excessive weight gains such as the genes, metabolism, diet, physical activity, and the influence of the wider social and cultural environment which characterizes 21st-century living [11]. Certain diseases such as Cushing syndrome and certain drugs such as beta-blockers also play a significant role in obesity [12, 13].

Extreme obesity is a global health problem and has acquired the level of a pandemic of chronic non-communicable disorder with multi-organ involvement [14]. The psychosocial impact of the condition can be quite distressing and troublesome, as patients face stigmatization and discrimination in many areas of their lives [15]. This may lead to depression and major mental health problems.

The phenomenon of obesity becomes a vicious circle as being overweight negatively impacts mobility, exercise, and other physical activities, and is associated with a mobility disability, hence the patient continues to gain weight. Childhood obesity has been very well linked to adulthood obesity [16]. Various single gene effects have been identified in severe early-onset obesity.

Extreme obesity is related to higher morbidity and mortality rates and is associated with multiple system disorders such as insulin resistance and hyperinsulinemia, type 2 diabetes, hypertension, dyslipidemia, coronary heart disease, gallbladder disease and cancer [17].

Losing weight and maintaining it is difficult. It definitely needs a lot of hard work and motivation. Regular exercise, a balanced diet, and a healthy lifestyle can definitely help in reducing weight. Weight loss drugs may be used but results are not uniform and satisfactory. Drugs can have side effects and patients usually regain weight after they stop taking these drugs.

In extreme obesity patients, it is almost impossible to get to normal weight range naturally i.e. by exercise and diet control. Hence other methods such as surgical procedures can definitely help in reducing the weight significantly [18]. Bariatric surgery has come a long way in helping this group of patients, but these techniques have their own side effects and complications. Only well-motivated patients should be counseled for such surgical procedures.

## Conclusions

The prevalence of overweight and extreme obesity has been increasing over the past two decades. Extreme obesity is associated with a substantially elevated mortality rate, due to DVT and pulmonary embolism. The number of bariatric surgical procedures performed annually is relatively small but increasing. Several risk factors for obesity are known, including a sedentary lifestyle, diet, increasing age, and low socioeconomic status. Identifying these risk factors can help physicians identify early which patients are at risk of developing extreme obesity. A healthy lifestyle along with a well-balanced diet, exercise, and a strong mental makeup is the mainstay to avoid or manage extreme obesity.

## References

[1] Must A, Spadano J, Coakley EH, Field AE, Colditz G, Dietz WH (1999). The disease burden associated with overweight and obesity. JAMA.

[2] Burton BT, Foster WR (1985). Health implications of obesity: an NIH Consensus Development Conference. J Am Diet Assoc.

[3] Hensrud DD, Klein S (2006). Extreme obesity: a new medical crisis in the United States. Mayo Clin Proc.

[4] Burwell CS, Robin ED, Whaley RD, Bickelmann AG (1956). Extreme obesity associated with alveolar hypoventilation—a Pickwickian syndrome. Am J Med.

[5] Martino JL, Stapleton RD, Wang M (2011). Extreme obesity and outcomes in critically ill patients. Chest.

[6] Syrakas CA, Neumaier-Prauser P, Angelis I, Kiask T, Kemkes BM, Gansera B (2007). Is extreme obesity a risk factor for increased in-hospital mortality and postoperative morbidity after cardiac surgery? Results of 2251 obese patients with BMI of 30 to 50. Thorac Cardiovasc Surg.

[7] Anandacoomarasamy A, Caterson I, Sambrook P, Fransen M, March L (2008). The impact of obesity on the musculoskeletal system. Int J Obes.

[8] Wearing SC, Hennig EM, Byrne NM, Steele JR, Hills AP (2006). Musculoskeletal disorders associated with obesity: a biomechanical perspective. Obes Rev.

[9] Kortt M, Baldry J (2002). The association between musculoskeletal disorders and obesity. Aust Health Rev.

[10] Klovaite J, Benn M, Nordestgaard BG (2015). Obesity as a causal risk factor for deep venous thrombosis: a Mendelian randomization study. J Intern Med.

[11] Wright SM, Aronne LJ (2012). Causes of obesity. Abdom Radiol.

[12] Al Suwaidi J, Higano ST, Hamasaki S, Holmes DR Jr, Lerman A (2001). Association between obesity and coronary atherosclerosis and vascular remodeling. Am J Cardiol.

[13] Galletti F, Fasano ML, Ferrara LA, Groppi A, Montagna M, Mancini M (1989). Obesity and beta‐blockers: influence of body fat on their kinetics and cardiovascular effects. J Clin Pharmacol.

[14] Roth J, Qiang X, Marbán SL, Redelt H, Lowell BC (2004). The obesity pandemic: where have we been and where are we going?. Obes Res.

[15] Wardle J, Cooke L (2005). The impact of obesity on psychological well-being. Best Pract Res Clin Endocrinol Metab.

[16] Forhan M, Gill SV (2013). Obesity, functional mobility and quality of life. Best Pract Res Clin Endocrinol Metab.

[17] Pi‐Sunyer FX (2002). The obesity epidemic: pathophysiology and consequences of obesity. Obes Res.

[18] Smith BR, Schauer P, Nguyen NT (2011). Surgical approaches to the treatment of obesity: bariatric surgery. Med Clin North Am.

